# Working towards a new psychiatry - neuroscience, technology and the DSM-5

**DOI:** 10.1186/1747-5341-7-1

**Published:** 2012-01-13

**Authors:** Sabina Alam, Jigisha Patel, James Giordano

**Affiliations:** 1 BioMed Central, Gray's Inn Road, London, WC1 8HB, UK; 2 Center for Neurotechnology Studies, Potomac Institute for Policy Studies, Arlington, VA 22203, USA; 3 Oxford Centre for Neuroethics/Oxford Uehiro Centre for Practical Philosophy, University of OxfordOxford, UK

## Abstract

This Editorial introduces the thematic series on '*Toward a New Psychiatry: Philosophical and Ethical Issues in Classification, Diagnosis and Care*' http://www.biomedcentral.com/series/newpsychiatry.

## Editorial

In the main, medicine has progressed substantially in recent years due to technological advances that have arguably enhanced methods to analyse research data, improved diagnostic techniques, and enabled more effective therapeutics. Such advancements are also being employed increasingly in psychiatry; in particular, progress in neuroimaging and other neurophysiological techniques, developments in behavioural sciences and psychotherapies, and developments in psychiatric genetics have all contributed to knowledge of mental illnesses. However, we must ask if, and in what ways these developments actually have affected the science, practice and clinical value of psychiatry. In addition, psychiatry is also confronting increasing diversity in socio-cultural values, norms and perspectives. The constructs of normality and abnormality, mental health and disorder, and the need for varying types of preventive and therapeutic interventions are undergoing iterative change. In part, this is reflected in, and influenced by the fifth edition of the *Diagnostic and Statistical Manual *(*DSM-5*) of the American Psychiatric Association, which upon release in spring of 2013, is hoped to instantiate greater consistency, and improve the ways that psychiatric disorders are classified and diagnosed [1], and lead to a greater conformity of psychiatric therapeutics. Yet, we must ask how these changes will affect the profession and practice on the world-stage, given an increasing trend toward pluralisation, and a need to address multi-cultural values and expectations about the nature of mental health and goals of psychiatric practice. Thus, we posit that the profession and practice of psychiatry is poised for evolution, and there is a need to review how current practices should be regarded, revised, implemented, and monitored.

Such re-assessment and revision will be important as worldwide social changes prompt new challenges and opportunities in psychiatric research and practice: international ethnic, religious and political beliefs and behaviours are becoming evermore prevalent, and the field is gaining prominence in non-western nations such as Japan, China and India. These changes necessitate reflection and insight in the philosophical and scientific bases of the profession, the ethical, legal, and social implications, as well as questions and problems that may be incurred through its articulation. Namely, how will - and perhaps should - advances in neuroscience, neurotechnology and genetics alter studies, concepts and the practise of psychiatry? In what ways might the DSM-5 affect the practical, ethical and legal aspects of the field, and its role in society? How might psychiatric diagnostic and therapeutic practices be best suited to meet the contingencies of non-Western societies? What specific constructs, paradigms, tools and techniques might shape and define this future path?

This thematic issue, '***Toward a New Psychiatry: Philosophical and Ethical Issues in Classification, Diagnosis and Care***', addresses these questions, concepts, problems and possible solutions from multi-disciplinary perspectives. The issue begins with a paper by Shadia Kawa and James Giordano that presents the historiography of the DSM and posits the implications of both the history of the DSM, and the potential of the DSM-5 to affect the current and future canon and practice(s) of psychiatry [2]. James Phillips and colleagues review what they suggest to be six essential questions in psychiatric diagnosis, and discuss how definitional issues in diagnostic schemas and concepts affect the scope and conduct of psychiatric care, and the social relevance of the field [3]. In response to proposed changes in the DSM-5, Morten Hesse discusses how revisions in the diagnostic criteria for antisocial personality disorder might lead to more accurate understanding, and improved therapeutics [4].

The use, utility and real value of the psychopharmacological (vs psychotherapeutic) approach to depression is addressed in a point-counterpoint dialectic provided by John Ioannidis, and John M Davis and colleagues, which then raises important questions about the actual nature of psychiatric disorder [5,6]. Indeed, this concept is the focus of ongoing debate, as Somogy Varga notes in offering a more naturally-based groundwork upon which to structure constructs of psychiatric norms, and deviation [7]. S. Nassir Ghamei offers a detailed discussion of what he refers to as "nosologomania", and questions trends toward excessive categorization in light of Karl Jaspers' critique of Emil Kraepelin's approach to classification - and diagnosis - of psychiatric states and conditions [8]. An essay by Randolph M Nesse and Dan J Stein addresses the validity of a genuinely medical model for psychiatric nosology [9], which relates to Tejas Patil and James Giordano's examination of core ontological assumptions of the medical model of psychiatry, and an attempt to re-align psychiatric canon with core features of this model that are sustainable through a bio-psychosocial orientation to constructs of neuroscience, normality, and culture [10]. In this vein, Peter Zachar and Kenneth S Kendler address the categorisation of homosexuality, and describe how changing constructs of science, society and medicine must be elucidated and acknowledged when ascribing social values to psychiatric nosologies, diagnoses and the ontological and socio-legal effects such claims manifest [11].

As a result of advancements in the neurosciences, studies of brain-behaviour relationships have increasingly become part of the psychiatric literature, as evidenced by the application of neuroimaging tools, such as functional magnetic resonance imaging (fMRI). But neurotechnological approaches, while important, are not without limitations, and like any tool, must be used in ways that are technically correct, ethically sound, and effective in practise. Kristina M Visscher and Daniel H Weissman argue that advances in cognitive neuroscience research can be facilitated by greater sharing of raw fMRI data between researchers, thereby allowing effective comparison, syntheses and validation of research-based information that can be translated into more meaningful clinical diagnostics and therapeutics [12]. Studies of neurocognitive developmental disorders, and addiction disorders, have also increased significantly in recent years, leading to the re-addressing treatment options to include novel approaches to cognitive behavioural, and pharmacological treatments. Kevin M Antshel and colleagues address recent advances in the understanding and treatment of Attention Deficit Hyperactivity Disorder (ADHD), and specifically discuss the use of both tailored formulations of stimulant drugs and cognitive behavioral interventions [13]. Daryl Shorter and Thomas R Kosten review recent pre-clinical studies and clinical trials in the treatment of cocaine addiction and emphasize the potential for future therapeutic developments [14]. Discussion of attention and addiction, and the use of psychoactive agents calls forth the proverbial "treatment-enhancement' debate, and Dan J Stein approaches this point through a thorough discussion of psychopharmacology and its potential to alter, affect and augment human cognition, emotion and behaviour [15].

While striving to define notions of technical rectitude, and individual and social good are crucial to the practise of any form of medicine, we must ask what new developments in the neurosciences, and changes in society portend for the future of psychiatry, and its place in contemporary cultures? Jennifer H Radden explicitly deals with such issues in her examination of patient rights, the nature, values and varied needs of mental health consumers, and the ways that reconstructive cultural semantics may influence clinical, ontological and socio-legal distinctions relative and relevant to psychiatric care [16]. Explicating problems, and limitations, and posing possible solutions is critical if psychiatry is to evolve as a consistently viable discipline in current and future society. Yet, implementing such change may require re-evaluation of philosophical, theoretical and economic impediments, as Nathan M Gerard elucidates [17]. To be sure, these factors figure prominently into any calculus that will determine the future of psychiatry. Yet, it is important to understand that shifting scientific, socio-economic, and even political realities will influence the trajectories that psychiatry can and will assume. Thus, as Alexander M Carson and Peter Lepping notes [18], it will be important to ground psychiatry to the ethical tenets of medicine - a practice in the literal sense, as an exchange of good, defined by those in relationship - that is dedicated to both the technically right and morally sound care of those patients that are the moral subject of clinical responsibility. It is in this spirit of better defining how science, technology and social forces will affect - and be affected by - psychiatry, both today and in the years to come.

The ideas and opinions detailed in these papers represent insights to the ways that changing conceptualisations of mental function, health, disorder and illness inform and direct the current and future practices of psychiatric research and treatment. We aim to continue to publish important articles which address these issues, and invite you to submit your manuscript to this cross-journal, thematic series (edited by James Giordano, Editor-in-Chief of *Philosophy, Ethics, and Humanities in Medicine*). Submissions addressing these themes are invited to the following journals for inclusion: *BMC Medicine; BMC Psychiatry; BMC Neuroscience; BMC Neurology; Genome Medicine *and *Philosophy, Ethics, and Humanities in Medicine*.

The constructs and practices of psychiatry (and the world stage upon which it which it will be enacted), will be explored further in a two-day conference: ***Towards a New Psychiatry: Implications of Neuroscience, Neurotechnology and the DSM-5***, which will be held in Washington, DC, USA, in April 2013 http://www.toward-a-new-psychiatry.com/. The conference will address these issues from multi-disciplinary perspectives, and will bring together researchers, scholars, and clinicians to engage in discussion, debate and dialectic about the historicity, canon, science, philosophy and ethics of psychiatry as profession and practice, and what the field can - and perhaps should - become.

## Authors' information

Sabina Alam is the Editor of *BMC Medicine *at BioMed Central. Jigisha Patel is the Medical Editor for *BMC-series journals *at BioMed Central. James Giordano is the Editor-in-Chief for *Philosophy, Ethics, and Humanities in Medicine*.
